# Overexpression of *RuFLS2* Enhances Flavonol-Related Substance Contents and Gene Expression Levels

**DOI:** 10.3390/ijms232214230

**Published:** 2022-11-17

**Authors:** Xin Huang, Yaqiong Wu, Shanshan Zhang, Hao Yang, Wenlong Wu, Lianfei Lyu, Weilin Li

**Affiliations:** 1 Institute of Botany, Jiangsu Province and Chinese Academy of Sciences (Nanjing Botanical Garden Mem. Sun Yat-Sen), Jiangsu Key Laboratory for the Research and Utilization of Plant Resources, Qian Hu Hou Cun No. 1, Nanjing 210014, China; 2 Co-Innovation Center for Sustainable Forestry in Southern China, Nanjing Forestry University, 159 Longpan Road, Nanjing 210037, China

**Keywords:** blackberry, flavonoids, molecular mechanism, flavonol synthase, metabolite

## Abstract

As an emerging third-generation fruit, blackberry has high nutritional value and is rich in polyphenols, flavonoids and anthocyanins. Flavonoid biosynthesis and metabolism is a popular research topic, but no related details have been reported for blackberry. Based on previous transcriptome data from this research group, two blackberry flavonol synthase genes were identified in this study, and the encoded proteins were subjected to bioinformatics analysis. RuFLS1 and RuFLS2 are both hydrophobic acidic proteins belonging to the 2OG-Fe(II) dioxygenase superfamily. RuFLS2 was expressed at 27.93-fold higher levels than RuFLS1 in red–purple fruit by RNA-seq analysis. Therefore, RuFLS2-overexpressing tobacco was selected for functional exploration. The identification of metabolites from transgenic tobacco showed significantly increased contents of flavonoids, such as apigenin 7-glucoside, kaempferol 3-*O*-rutinoside, astragalin, and quercitrin. The high expression of RuFLS2 also upregulated the expression levels of NtF3H and NtFLS in transgenic tobacco. The results indicate that RuFLS2 is an important functional gene regulating flavonoid biosynthesis and provides an important reference for revealing the molecular mechanism of flavonoid accumulation in blackberry fruit.

## 1. Introduction

Blackberry (*Rubus* spp.) is a perennial shrub to small tree in the genus *Rubus*, family Rosaceae, that produces small berries [1]. Blackberry is native to North America and has a long history of cultivation in Europe and the United States. In 1986, blackberry was first introduced into China and popularized by the Institute of Botany, Chinese Academy of Sciences in Jiangsu Province. It has the advantages of drought resistance, vigorous growth, and easy reproduction [2,3]. The fruits are rich in nutrients such as sugars, vitamin C, vitamin E, polyphenols, and flavonoids [4,5] and have extremely high nutritional and medicinal value, including antibacterial, anti-inflammatory, antioxidant and antiaging effects [6,7]. These fruits are widely used in medical and health care fields with good prospects for further applications.

Flavonoids are a class of plant-produced secondary metabolites belonging to the group of polyphenolic compounds with a typical C6-C3-C6 carbon skeleton [8]. Flavonoids can be divided into six subclasses, including flavonoids, isoflavones, dihydroflavonoids, flavonols, flavonones, and anthocyanins [9]. Flavonols can be modified by acylation, hydroxylation, and glycosylation. These modifications can coexist, resulting in diverse flavonol structures [10], so these compounds have a variety of biological activities, such as antioxidation, antiviral, and anticancer activities [11,12,13]. In addition, these compounds can aid in resistance to ultraviolet radiation damage [14], enhance the ability of plant roots to resist drought stress [15], and play an important biological role in plant growth and development. Thus, flavonols are among the important substances that participate in plant resistance to external biotic and abiotic stresses.

Flavonol synthase (FLS) is one of the key enzymes in the biosynthetic pathway of flavonoids. The *FLS* genes play an important role in the appearance of white or yellow plant organs, and the overexpression of the *Muscari aucheri FLS* gene resulted in a white flower color [16]. Plant FLS proteins are often encoded by multiple gene copies, and the number of copies is different in different plants [17]. The expression level of *FLS* has significant spatial and temporal differences in different tissue parts, metabolic stages, and growth and developmental stages of plants [18], which affects the content of flavonoids in plants. *FLS* genes have been identified and functionally studied in some plants, with similar functions found in most plants. For example, OcFLS1 and OcFLS2 of *Ornithogalum caudatum* can both catalyze the production of flavonol from dihydroflavonol, and both have flavanone 3-hydroxylase (F3H) activity [19], while several FLSs in some plants have different functions. In *Arabidopsis thaliana*, FLS1 is involved in the biosynthesis of flavonoids, while FLS3 catalyzes the production of flavonols [20]. Therefore, although FLS is involved in the synthesis of flavonols, its expression pattern and specific regulatory mechanism in blackberry still need to be further studied and verified.

In this study, based on the transcriptome data of immature and mature blackberry fruits obtained previously by this research group [21], the *FLS* gene showed significant differential expression in the flavonoid synthesis pathway and was selected for further research. Its effect on flavonoid synthesis in blackberry fruit was investigated along with the effects of related substances. The purpose of this study was to (i) identify, clone and bioinformatically analyze the potential functions of *FLSs*; (ii) analyze the expression patterns of differentially expressed *FLSs* in different tissues and fruit developmental stages in blackberry; and (iii) study the function of *FLS* overexpression and explore its effects on the accumulation of related metabolites and gene expression to provide a basis for the study of flavonoid biosynthesis and FLS function in blackberry fruit.

## 2. Results

### 2.1. Gene Structure, Physicochemical Properties and Domains of RuFLSs

**Figure 1:**
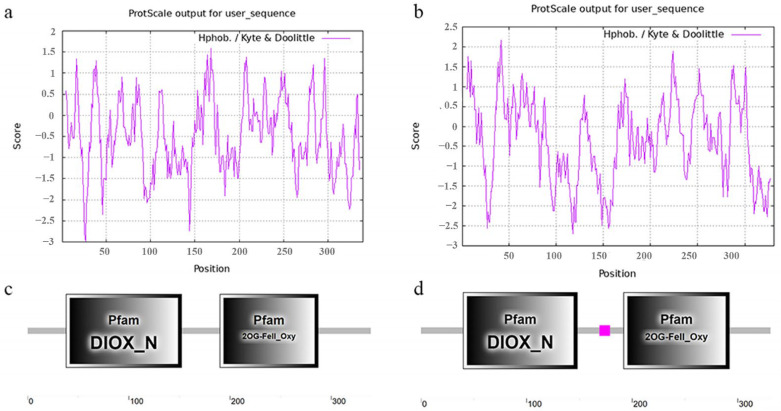
Prediction of the hydrophilicity and structural domains of RuFLS proteins. (**a**) Hydrophilicity of RuFLS1; (**b**) Hydrophilicity of RuFLS2; (**c**) Structural domains of RuFLS1; and (**d**) Structural domains of RuFLS2.

From the transcriptome data of blackberry at different developmental stages (SRA: PRJNA701162) [21], we found three genes that may encode FLS proteins: one gene did not contain a verified ORF, while the other two genes are named RuFLS1 and RuFLS2. Structural analysis showed that the ORF sequences of RuFLS1 and RuFLS2 were 1017 and 993 bp in length, respectively. The physicochemical properties of RuFLS1 and RuFLS2 proteins were analyzed by ProtParam software (SIB, Geneva, Switzerland), including protein molecular weight, theoretical isoelectric point, charged residue number, instability index, fat index, and hydrophilicity (Table 1). The instability index was 36.45 and 34.70, and the instability coefficient was less than 40; thus, both proteins were considered to be stable. In addition, the total hydrophilicity of RuFLS1 and RuFLS2 was predicted to be −0.494 and −0.341 (Table 1), respectively, by ProtScale online software (SIB, Geneva, Switzerland) (Figure 1a,b), suggesting that both proteins are hydrophobic and acidic. The functional domains of RuFLS1 and RuFLS2 were predicted and found to contain a DIOX_N functional domain and a 2OG-FeII_Oxy functional domain (Figure 1c,d), indicating that they belong to the α-oxoglutarate-dependent dioxygenase family. The DIOX_N functional domain of RuFLS1 is located at amino acids 38–152, and the 2OG-FeII_Oxy functional domain is located at amino acids 190–287. The DIOX_N functional domain of RuFLS2 is located at amino acids 40–148, and the 2OG-FeII_Oxy functional domain is located at amino acids 192–292. The positions of the functional domains of different members are not exactly the same. RuFLS1 and RuFLS2 have no transmembrane region and are both extramembrane proteins.

### 2.2. Multiple Sequence Alignment, Phylogenetic Analysis, and Motif Composition of RuFLSs

**Figure 2:**
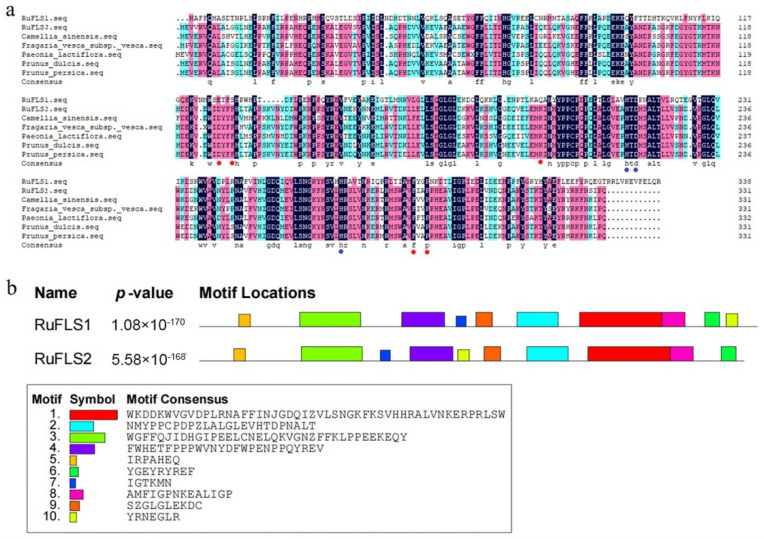
Multiple sequence alignment and motifs of FLS amino acid sequences. (**a**) Multiple sequence alignment of FLS amino acid sequences in different plants. (**b**) Conserved domains or motifs in the RuFLSs.

Amino acid alignment of the FLS protein sequences of multiple plants by DNAMAN software (Lynnon Biosoft, California, USA) showed that RuFLS1 and RuFLS2, as well as sequences from *Prunus dulcis* (XM_034342566.1), *Prunus persica* (XM_007222519.2), *Paeonia lactiflora* (KM259902.3), *Fragaria vesca* subsp. *Vesca* (XM_011460471.1) and *Camellia sinensis* (DQ198089.1) were highly conserved. In the figure, the red dots indicate important amino acid residues that bind to the substrate dihydroquercetin, and the blue dots indicate amino acid residues that are important for binding to ferrous ions (Figure 2a). Interestingly, RuFLS1 was mutated at multiple substrate-binding sites and may have lost part of its protein function during blackberry evolution. Differential gene expression analysis based on blackberry transcriptome data showed that RuFLS1 was not differentially expressed, which may have been related to the changes in amino acid residues at key sites of the RuFLS1 protein rather than in key genes regulating important secondary metabolites such as flavonoids. RuFLS2 is a significantly differentially expressed enzymatic gene, and it is speculated that this gene may play an important role in the accumulation of flavonoids during blackberry fruit ripening. Therefore, we conducted an in-depth study on the function of RuFLS2 to reveal the mechanism by which RuFLS2 mediates the synthesis and metabolism of flavonoid-related secondary metabolites in blackberry.

The conserved motifs of the two RuFLS proteins were predicted. The results showed that both RuFLS1 and RuFLS2 had eight identical conserved motifs, and their positions within the sequence were basically the same (Figure 2b). The green part in the figure is the DIOX_N superfamily, a highly conserved N-terminal region of proteins with 2-oxoglutarate/Fe(II)-dependent dioxygenase activity. The blue area is the Fe(II)-dependent oxygenase superfamily, and the red area is the 2OG superfamily. The latter two regions combine to form the 2OG-Fe(II)_oxygenase superfamily motif, which is consistent with the predicted domain and the FLS protein family in plants. Two consistently conserved motifs, “IGTKMN” and “YRNEGLR”, are arranged in different positions in RuFLS1 and RuFLS2, which may lead to differences in the functions of these proteins.

**Figure 3:**
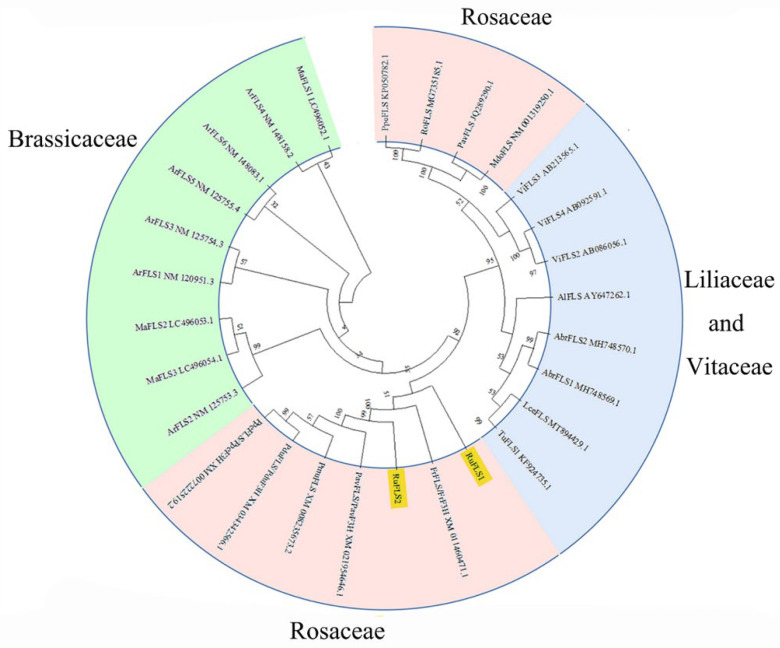
Phylogenetic tree of blackberry RuFLSs (RuFLS1 and RuFLS2) and FLS genes from other species. The rings of different colors represent different genera.

A phylogenetic tree was constructed with MEGAX with the NJ method. The results showed that RuFLS1 and RuFLS2 were closely related to flavonol synthase/flavanone 3-hydroxylase proteins from Rosaceae species such as *Fragaria vesca* subsp. *vesca*, *Prunus avium*, *Prunus dulcis*, *Prunus persica*, and *Prunus mume*. The flavonol synthases of Brassicaceae and the flavonol synthases of Liliaceae and Vitaceae are clustered into a branch, which is in line with the relationship based on species evolution (Figure 3). Both are Rosaceae plants, and the relationship between flavonol synthase proteins in *Prunus persica*, *Malus domestica*, and *Prunus avium* with RuFLS1 and RuFLS2 is distant. It is speculated that RuFLS1 and RuFLS2 are not monofunctional flavonol synthase enzymes but may be bifunctional enzymes with both FLS and F3H activities.

### 2.3. Expression Levels of RuFLSs in Different Tissues and Fruit Developmental Stages of Blackberry

**Figure 4:**
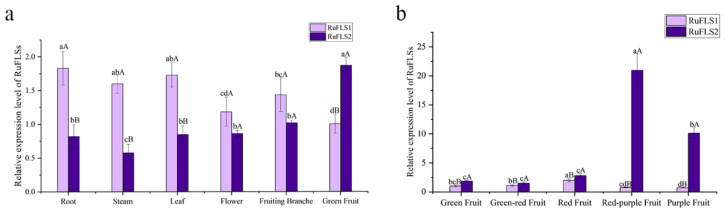
The expression pattern of the *RuFLS* genes in different tissues (**a**) and different developmental stages of blackberry fruits (**b**). Different lowercase letters indicate significant differences between different harvest periods (*p* < 0.05). Different uppercase letters represent significant differences between two *RuFLS* genes (*p* < 0.05).

We analyzed the expression levels of *RuFLS1* and *RuFLS2* in the roots, stems, leaves, flowers, fruiting branches and fruits of blackberry and found that the expression level of *RuFLS2* was higher in fruits, while the expression level of *RuFLS1* was higher in stems and leaves. In comparison, the expression level of *RuFLS1* in fruits was significantly lower than that of *RuFLS2*, but it was higher in other tissues. It was speculated that *RuFLS2* might play a major role in blackberry fruits (Figure 4a). Based on this, we also tested the expression level of the *RuFLS* genes at different fruit developmental stages and found that the expression level of *RuFLS1* was significantly lower than that of *RuFLS2* at each fruit developmental stage. The *RuFLS2* gene showed more significant differences during fruit development. The expression level of *RuFLS2* increased sharply as the color of the fruit changed from red to purple. It then decreased rapidly but was still higher than that in the early stage of fruit development (Figure 4b).

### 2.4. Changes in Flavonoid, Phenol, and Anthocyanin Contents in Blackberry Fruits at Different Developmental Stages

To gain an in-depth understanding of the changes in flavonoid-related substances during the development of blackberry fruits, we also measured the contents of total phenols, flavonoids and anthocyanins in blackberry fruits at different stages. The results showed that the content of total phenols and flavonoids changed in a similar way, showing a sharp decline in the early stage and little change in the later stage. The content increased slightly in the purple fruit stage, and the content in the green fruit stage was significantly higher than that in the other stages. The total phenolic content reached 40.68 mg·g^−1^ FW, and the flavonoid content reached 11.50 mg·g^−1^ FW. The accumulation of anthocyanins in the early stage of fruit development was lower and tended toward zero, and the amount of anthocyanins increased sharply from the red and purple fruit stage to the purple fruit stage, reaching a maximum of 2.60 mg·g^−1^ FW at the purple fruit stage. This result indicated that anthocyanins are mainly synthesized and accumulate in the later stage of fruit development (Table 2).

### 2.5. Heterologous Overexpression of RuFLS2 in Tobacco

**Figure 5:**
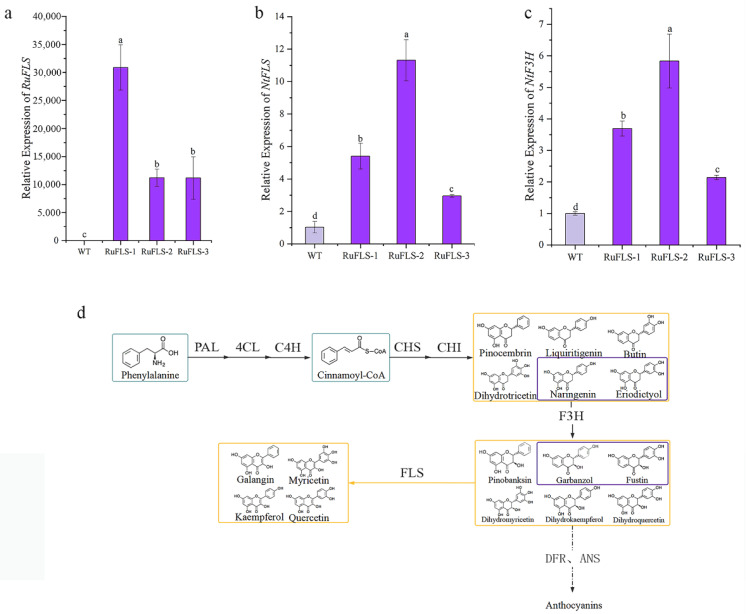
Expression patterns of flavonoid biosynthesis pathway genes in WT and RuFLS2-overexpressing tobacco. (**a**) *RuFLS2* gene; (**b**) *NtFLS* gene; and (**c**) *NtF3H* gene. Significant differences between means (*p* < 0.05) are indicated by lowercase letters (a, b, c and d) above the bars. (**d**) Biosynthetic pathway of flavonoid compounds in blackberry. WT represents wild-type tobacco (*Nicotiana tabacum* cv. Wisconsin 38). RuFLS2-1, RuFLS2-2 and RuFLS2-3 represent three different transgenic tobacco lines.

To reveal the biological functions of *RuFLS2*, we overexpressed *RuFLS2* in tobacco. Three transgenic lines with relatively vigorous growth were randomly selected for expression level detection, and it was found that *RuFLS2* was highly expressed in the transgenic plants (Figure 5a). To explore the effect of *RuFLS2* heterologous expression on key enzyme genes in the tobacco flavonoid biosynthesis pathway, we detected the expression level of tobacco *NtFLS* and the upstream gene *NtF3H* (Figure 5d). The results showed that the expression level of the *NtFLS* gene in transgenic tobacco was significantly increased (Figure 5b), and the change trend of the expression level of the *F3H* gene in transgenic tobacco across the three lines was consistent with that of *FLS* (Figure 5c). The upregulation of *NtFLS* and *NtF3H* in the RuFLS-1 and RuFLS-2 lines was significantly higher than that in RuFLS-3. The results confirmed that heterologous expression of *RuFLS2* upregulated the expression of the tobacco *NtFLS* gene.

### 2.6. Flavonoid and Phenol Content Analysis in Genetically Modified Tobacco

**Figure 6:**
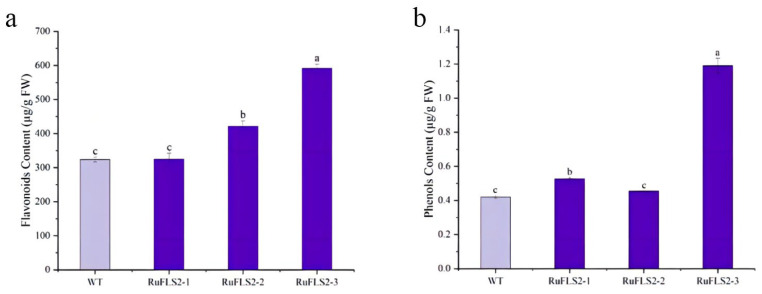
Flavonoid (**a**) and phenol (**b**) contents in WT and RuFLS2-overexpressing tobacco. WT represents wild-type tobacco. RuFLS2-1, RuFLS2-2 and RuFLS2-3 represent three different transgenic tobacco lines. Significant differences between means (*p* < 0.05) are indicated by lowercase letters (a, b and c) above the bars.

To analyze the effect of overexpression of the *RuFLS2* gene on flavonoid-related metabolites, the contents of flavonoids and total phenolics in the leaves of the transgenic FLS and WT lines were determined. The results showed that the flavonoid content of one transgenic tobacco line was the same as that of the WT, and the content of the other two lines increased by 1.3- and 1.8-fold (Figure 6a). The content of total phenol was similar to that of flavonoids, where one line had the same content as the WT, and the other two lines showed increases of 1.25- and 2.84-fold (Figure 6b). Overall, the overexpression of the *RuFLS2* gene increased the contents of flavonoids and phenols in tobacco, indicating that the overexpression of *RuFLS2* increased the contents of flavonoid-related substances in plants. As there are many kinds of flavonoids in plants, a metabolome analysis needs to be performed in the future.

### 2.7. Multivariate Statistical Analysis of Metabolites in Genetically Modified Tobacco

**Figure 7:**
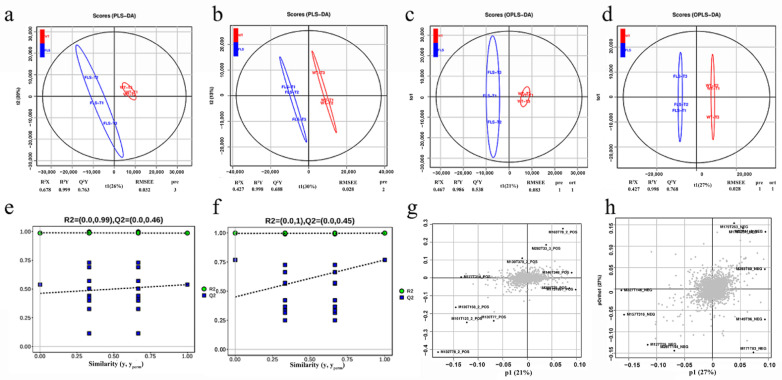
PLS-DA and OPLS-DA of WT and RuFLS2-OX tobacco. PLS-DA analysis of WT and RuFLS2-OX tobacco with positive (**a**) and negative (**b**) patterns. OPLS-DA of WT and RuFLS2-OX tobacco with positive (**c**) and negative (**d**) patterns. The OPLS-DA displacement test of WT and RuFLS2-OX tobacco with positive (**e**) and negative (**f**) patterns. Loading plot diagram of WT and RuFLS2-OX tobacco with positive (**g**) and negative (**h**) patterns.

PLS-DA can maximize the distinction between groups and is beneficial for finding differentially expressed metabolites (DEMs). This study showed that the tobacco metabolites in the WT control group and the FLS transgenic group were R^2^X = 0.678, R^2^Y = 0.999, and Q^2^Y = 0.763 in positive ion mode (Figure 7a); in negative ion mode, they were R^2^X = 0.427, R^2^Y = 0.998, and Q^2^Y = 0.688 (Figure 7b), in line with our expectations for the model of the experimental data. These results indicate that the parameters of the PLS-DA model established in this study were reasonable and stable and could be used for metabolomic analysis. OPLS-DA is a derivative algorithm of PLS-DA (Figure 7c,d). Both multivariate statistical analyses of supervised pattern recognition found that the FLS group was on the left side of the confidence zone, while the WT group was on the right side of the confidence zone, indicating that both models can effectively distinguish the metabolites of transgenic and nontransgenic tobacco. In addition, we validated the OPLS-DA model by permutation and found that the OPLS-DA model had good reliability (Figure 7e–h), and the subsequent model checking and differential metabolite screening were analyzed using the OPLS-DA results.

### 2.8. DEM-Related Flavonoid Synthesis in Tobacco

To further explore the effect of overexpression of the *RuFLS2* gene on flavonoid-related metabolites, we focused on the analysis of DEMs related to flavonoid synthesis in the transgenic FLS group and WT group. The results showed that six DEMs were detected in the transgenic tobacco flavonoid biosynthesis pathway, and the contents of the other five substances, except naringenin-7-*O*-glucoside, were decreased. A total of seven DEMs were detected in the flavone and flavonol synthesis pathways, among which the contents of apigenin 7-glucoside, kaempferol 3-*O*-rutinoside, astragalin and quercitrin were significantly increased, while the contents of luteolin 7-glucoside, quercetin 3-glucoside and ligustroflavone were decreased (Table 3). This result indicated that the overexpression of the *RuFLS2* gene consumed upstream flavonoids and significantly increased the flavonol content in plants.

## 3. Discussion

As a third-generation fruit, blackberry is rich in a variety of flavonoids and has high research value [22]. However, research progress on the enzymatic genes related to flavonoid synthesis in blackberry is still lacking. In this study, we identified two members of the *FLS* gene family from the blackberry transcriptome, named RuFLS1 and RuFLS2, and performed a bioinformatics analysis on them. The results showed that the amino acid sequences of the RuFLS1 and RuFLS2 proteins are very similar; both contain conserved functional domains, but RuFLS1 is mutated at multiple substrate binding sites, which may result in the loss of some protein functions. Relevant studies have shown that in plants, only some members of the multicopy gene family have functions [23]. For example, the *FLS* gene family in *Arabidopsis* contains six members, but only FLS1 can affect the biosynthesis of flavonoids [24]. Differential gene expression analysis based on blackberry transcriptome data showed that *RuFLS2* was a significantly differentially expressed enzymatic gene. Therefore, we performed preliminary screening and speculated that the FLS protein that mainly functions during blackberry fruit ripening may be RuFLS2.

To understand the evolutionary relationship between RuFLS1, RuFLS2 and FLSs of other species, the phylogenetic tree (Figure 3) showed that RuFLS2, RuFLS1 and the flavonol synthase/flavanone 3-hydroxylase proteins of other Rosaceae plants, such as *Fragaria vesca* subsp. *Vesca*, *Prunus avium*, and *Prunus persica*, are closely related. We speculated that blackberry RuFLS2 may have some F3H functions in addition to FLS functions. In the flavonoid biosynthesis pathway, F3H catalyzes the production of flavanones to dihydroflavonols, while FLS is located downstream of F3H and catalyzes the production of flavonols using dihydroflavonols as substrates (Figure 5). Both FLS and F3H belong to the 2OG-Fe(II)_oxygenase superfamily and have functional similarities [25]. Several experiments have shown that FLS is a bifunctional dioxygenase with F3H activity and FLS activity [26]. FLS extracted from *Citrus unshiu* can not only convert dihydrokaempferol to kaempferol but also directly use pomelo. Cortexin is a substrate used in the efficient production of kaempferol. Two FLSs isolated from *Ornithogalum caudatum* also have similar functions. Fe^2+^ is not necessary for F3H activity, but OcFLS directly catalyzes flavanones to flavonols and requires a sufficient amount of Fe^2+^ to participate in the reaction [27].

The expression of the *FLS* gene has obvious spatial and temporal differences. *FLS* is mainly highly expressed in the petals of ornamental plants [28] and regulates changes in flower color. In economic fruit trees, such as grapes, the expression levels and enzyme activities of FLSs are highly positively correlated with the content of flavonoids in the fruit, and the expression level is higher at the veraison and ripening stages of grapes. In this study, it was found that *RuFLS1* was highly expressed in roots, stems and leaves. The expression level of *RuFLS2* in blackberry fruit was significantly higher than that in other tissues. During the development of blackberry fruit, the expression level of *RuFLS1* was always lower than that of *RuFLS2*. The expression level of *RuFLS2* was lower in the early stage of fruit development and was higher when the fruit epidermis developed a red–purple color. The expression level increased sharply during the red–purple period and decreased rapidly when the fruit turned dark purple and became fully ripened, which was consistent with the changes in the expression level of *FLS* in grapes [29].

To further explore the potential function of the differentially expressed gene *RuFLS2* in plants, we constructed an overexpression vector and successfully overexpressed the *RuFLS2* gene heterologously in tobacco. We examined the contents of flavonoids and phenols in transgenic tobacco. The results showed that overexpression of *RuFLS2* significantly increased the contents of flavonoid-related substances in transgenic tobacco. Based on these results, we performed a metabolomic analysis to further clarify the function of *RuFLS2*. The content of metabolites in the flavonol synthesis pathway in transgenic tobacco was significantly increased, consistent with the experimental results of Park et al. [30]. The heterologous expression of *AcFLS* in *Allium cepa* significantly increased the flavonol content in tobacco. Our study also found that the high heterologous expression of *RuFLS2* significantly increased the expression of the *NtFLS* and *NtF3H* genes in transgenic tobacco lines. *NtF3H*, as the upstream gene of *NtFLS*, was negatively regulated by *NtFLS*, and its expression level increased. Further investigation suggested that the biosynthesis of flavonoids was regulated not only by structural genes such as *F3H* and *FLS,* but also by transcription factors. The regulatory mechanism of flavonol biosynthesis was initially studied in Arabidopsis [31]. The MYB transcription factor regulates the expression of *FLS* to control the flavonol content in plants. Overexpression of *AtMYB12* can significantly increase the flavonoid content of *Arabidopsis thaliana*, and the flavonoid content of the *AtMYB12* mutant is significantly reduced [32]. Related studies have also found that apple *MdMYB22* [33] can directly bind to the promoter of *FLS* and activate its expression, and overexpression of pear *PbMYB12b* can significantly increase the synthesis and accumulation of quercetin glycosides [34], indicating that the transcription factor *MYB* plays an important role in flavonoid synthesis. In addition to the MYB transcription factor family, other transcription factor families, such as bZIP and WRKY [35], are also involved in the regulation of flavonoid biosynthesis. In short, the regulatory mechanism of flavonoid biosynthesis is very complex. To explore the molecular mechanism of flavonoid synthesis in blackberry, it is necessary to further study its transcriptional regulation.

## 4. Conclusions

In this study, we successfully identified and isolated the *RuFLS2* gene containing a 993-bp ORF. The *RuFLS2* gene encodes a hydrophobic acidic protein composed of 331 amino acids belonging to the 2OG-Fe(II)_oxygenase superfamily. A temporal and spatial expression analysis showed that *RuFLS2* was highly expressed in blackberry fruit, and the expression peaked when the fruit developed a red–purple color. Heterologous overexpression in tobacco revealed that the synthetic content of flavonols, such as apigenin 7-glucoside, kaempferol 3-*O*-rutinoside, astragalin, and quercitrin, increased; additionally, the high expression of *RuFLS2* increased the gene expression levels of *NtF3H* and *NtFLS* in transgenic tobacco. The results indicated that *RuFLS2* is a key enzymatic gene regulating the synthesis of flavonoids in blackberry and can affect the expression of other key structural genes in the flavonoid synthesis pathway, providing a new direction for studying flavonoid biosynthesis and its regulatory mechanisms.

## 5. Material and Methods

### 5.1. Plant Materials and Growth Conditions

**Figure 8:**
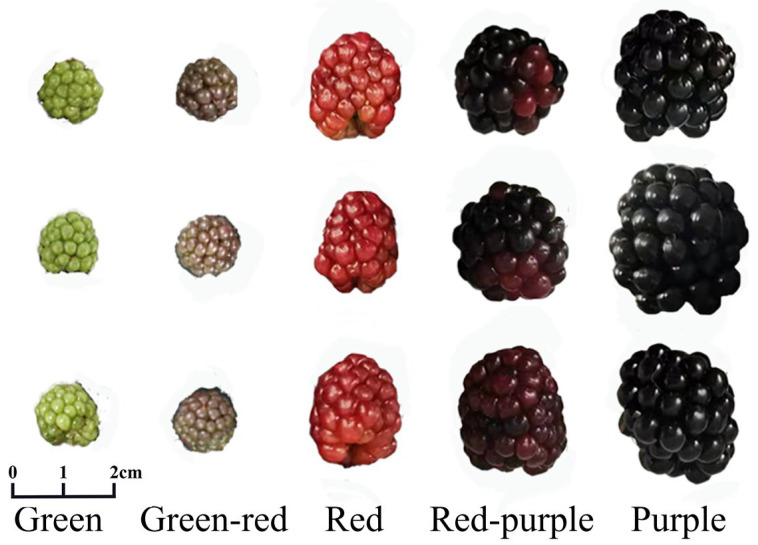
Appearance of blackberry cv. ‘Chester’ fruits at various developmental stages. According to the color of the fruit, the development of the fruit can be divided into five stages: green fruits, green–red fruits, red fruits, red–purple fruits, and purple fruits.

The experimental material was the blackberry cultivar ‘Chester’ from the Baima Experimental Base in Lishui District, Nanjing City (119°11′ E, 31°36′ N). The study area belongs to the subtropical monsoon climate zone. Specific growth conditions are listed in Supplementary Table S1. The roots, stems, leaves, flowers, fruiting branches and fruits of blackberry were picked as plant materials for the analysis of gene expression in different tissue parts. In addition, the fruit developmental stages were distinguished according to the color of the fruit. Green fruits, green–red fruits, red fruits, red–purple fruits and purple fruits were picked (Figure 8). All experiments were biologically repeated at least three times. After collection, samples were stored in liquid nitrogen and brought back to the laboratory for storage in a −80 °C refrigerator.

### 5.2. Total RNA Extraction and cDNA Reverse Transcription

Total RNA was extracted using a general plant total RNA rapid extraction kit (BioTeKe, Beijing, China). Total RNA from different fruit parts and developmental stages was reverse transcribed to synthesize cDNA. A subsequent expression analysis was performed using cDNA diluted to a concentration of approximately 300 ng/µL as a template.

### 5.3. Cloning of the RuFLS Gene

Based on the differentially expressed *RuFLS* unigene sequences identified in the blackberry transcriptome data, the open reading frame (ORF) and third-generation full-length transcript sets were obtained by BioXM2.6 software (Ji Feng, Nanjing, China) analysis for correction, and primers were designed with Oligo 6.0 software (Reachsoft, Beijing, China). The ORF sequence was cloned using the high-fidelity PCR enzyme PrimeSTAR Max DNA Polymerase (TaKaRa, Dalian, China). A 50 μL PCR system was utilized as follows: 25 μL of Primer Star Max, 1 μL of forward and reverse primers, 1 μL of cDNA template, and 22 μL of ddH_2_O. The PCR program was as follows: 98 °C for 3 min; 35 cycles of 98 °C for 10 s, 55 °C for 5 s, and 72 °C for 15 s; 72 °C for 3 min; and a 4 °C hold. The amplified product was ligated to the vector according to the requirements of the pClone007 Blunt Vector Kit (TSINGKE, Nanjing, China) and transferred to competent *Escherichia coli* cells; the potential positive bacteria were cultured at 37 °C and 250 rpm for 6–8 h on a shaker with 2 × T5 Super PCR Mix (Colony) (TSINGKE, Nanjing, China) and used for bacterial liquid PCR verification. The positive bacterial liquid was sent to Tsingke Biotechnology Co., Ltd. for first-generation Sanger sequencing verification. Primer sequences are listed in Supplementary Table S2.

### 5.4. Bioinformatics Analysis

The protein sequences encoded by the blackberry *FLS* genes were analyzed using DNAMAN software, and their physical and chemical properties and protein hydrophilicity were predicted using ExPASy software (SIB, Geneva, Switzerland) (https://web.expasy.org/, accessed on 19 February 2022). The MEME online tool (https://meme-suite.org/meme/tools/meme/, accessed on 24 March 2022) was used to predict the motifs of RuFLS proteins. The SMART online tool (http://smart.embl-heidelberg.de/, accessed on 19 February 2022) and NCBI online tool CDART (Conserved Domain Architecture Retrieval Tool) (https://www.ncbi.nlm.nih.gov/Structure/cdd/wrpsb.cgi#opennewwindow/, accessed on 19 February 2022) were used to analyze the domains of RuFLSs, including transmembrane domains, functional domains and conserved structure areas. In addition, RuFLS protein sequence alignment (similarity) was performed using MEGAX (MEGA, AZ, USA), and a phylogenetic tree was constructed by the neighbor-joining with 1000 bootstrap replicates.

### 5.5. qRT–PCR Analysis

qRT-PCRs were performed with TB Green Premix Taq II (Tli RNASEH Plus) (Takara, Dalian, China). A 15 μL reaction system was used as follows: 7.5 μL of TB Green Premix Taq II fluorescent dye, 1 μL of cDNA template, 0.6 μL of upstream and downstream primers, and 5.3 μL of ddH2O. The qRT–PCR program was as follows: 95 °C for 2 min; 40 cycles of 95 °C for 10 s, 60 °C for 10 s, and 72 °C for 15 s; and melting curve generation for 6 s. For blackberry, 18S was the internal reference gene (Supplementary Table S2), and for transgenic tobacco seedlings, NtActin was the internal reference gene (Supplementary Table S2) for real-time quantitative PCR. The 2^−ΔΔCT^ method was used for quantitative data analysis.

### 5.6. Determination of Polyphenol, Flavonoid and Anthocyanin Contents

The total phenol content was determined according to the method of Cheok et al. [36] using a commercial plant total phenol test kit (Nanjing Jiancheng Institute of Bioengineering, Nanjing, China). Fresh samples were ground into powder with liquid nitrogen. Then, 0.1 g of powder was taken, added to 2 mL of extract (60% ethanol aqueous solution), vortexed and mixed for 3 min, extracted at 60 °C for 30 min, and centrifuged at 4000 rpm·min^−1^ for 10 min. The supernatant was transferred to a centrifuge tube, with reagents added as needed, mixed, and allowed to stand at room temperature for 10 min. The absorbance of each tube was measured at 760 nm. The hydromethanolic gallic acid solution was freshly prepared in a series of concentrations (0.1–1 mM) and tested in parallel to establish a calibration curve. The total phenolic content of the samples was calculated as mg of gallic acid equivalent per g fresh sample (mg/g FW).

The flavonoid content was detected by the aluminum chloride calorimetry method [37] using a commercial plant flavonoid test kit (Nanjing Jiancheng Institute of Bioengineering, Nanjing, China). Samples were washed with normal saline, dried, ground with liquid nitrogen to powder, weighed to 0.05 g and combined with 2 mL of 60% ethanol. The solution was shaken, extracted at 60 °C for 2 h, and centrifuged at 10,000 rpm for 10 min at room temperature. The supernatant was collected for testing. According to the standard curve drawing procedure, each sample was tested three times, and the mean value was taken and substituted into the standard curve to calculate the flavonoid content, which was expressed as mg per g of FW (mg/g FW).

The anthocyanin content was determined by the pH differential method [38] with a slight modification: 50 g of blackberry fruit homogenate was taken, 3 g of homogenate solution was added, 30 mL of 50% ethanol extract was added, and the mixture was shaken well and ultrasonically treated at 60 Hz for 20 min at 35 °C. The mixture was then centrifuged at 5000 rpm for 5 min; then, 0.5 mL of the supernatant was taken and placed in a 10 mL centrifuge tube, 4.5 mL of pH 1.0 buffer was added, and the mixture was reacted at room temperature for 20 min and adjusted to zero with double distilled water. The absorbance was measured at 510 nm. A standard curve was utilized to calculate the anthocyanin content. The equation obtained for the calibration curve of the cyanidin-3-glucoside standard (C832095-5mg) (Nanjing Shoude Biotechnology Co., Ltd., Nanjing, China) solution in the range of 0–0.6 mg/mL was y = 0.0455x − 0.0881 (R^2^ = 0.9996). The results are expressed as mg per g of FW (mg/g FW).

### 5.7. qRT–PCR Analysis

The Agrobacterium culture containing the overexpression vector with the target fragment was expanded to 50 mL in liquid culture containing kanamycin (Solarbio, Beijing, China) and rifampicin (Solarbio, Beijing, China) antibiotics at 28 °C. The OD value of the bacterial culture was measured and maintained at 0.5–0.8. The bacterial solution was transferred to a 50 mL centrifuge tube and centrifuged at 5000 rpm for 10 min, and the supernatant was then discarded. The bacterial pellet was resuspended in 40 mL of MS0 (4.43 g/L MS (Beijing XMJ Scientific Co., Ltd, Beijing, China) + 100 μmol/L acetosyringone (Solarbio, Beijing, China), pH 5.8). The leaves of “*Nicotiana tabacum* cv. Wisconsin 38” that grew vigorously were collected and cut into small (1 cm^2^) pieces. The small leaf pieces were immersed in the prepared bacterial solution for 6–12 min and shaken several times during this period. The leaf pieces were spread on MS1 medium (4.43 g/L MS + 25 g/L sucrose + 5.6 g/L agarose (Solarbio, Beijing, China) + 2.0 mg/L 6-BA (Solarbio, Beijing, China) + 0.1 mg/L NAA (Solarbio, Beijing, China) + 100 μmol/L acetosyringone, pH 5.8) and cultured at 25 °C for 2–3 d in the dark. They were then transferred to MS2 medium (4.43 g/L MS + 25 g/L sucrose + 5.6 g/L agarose + 2.0 mg/L 6-BA + 0.1 mg/L NAA + 100 mg/L Timentin (Solarbio, Beijing, China) + 100 mg/L kanamycin, pH 5.8), placed under a 16 h light/8 h dark cycle, and cultured at 25 °C until shoots formed on the callus. The shoots were excised and transferred to MS rooting medium (4.43 g/L MS + 25 g/L sucrose + 5.6 g/L agarose + 100 mg/L Timentin, pH 5.8). The leaves were collected to extract RNA, which was reverse transcribed into cDNA when the transgenic plants showed strong growth. PCR cloning was performed with ORF-specific primers to verify the positive cloned plants.

### 5.8. LC–MS/MS Analysis of Tobacco

After thawing six tobacco samples at 4 °C, precooled methanol/acetonitrile (Merck, Darmstadt, Germany) /water solution (2:2:1, *v*/*v*) was added, followed by low-temperature ultrasonic extraction; the supernatant was then vacuum-dried. For mass spectrometry analysis, 100 μL of acetonitrile water solution was added (acetonitrile:water = 1:1, *v*/*v*) for reconstitution, and the supernatant was injected for analysis.

The samples were separated using an Agilent 1290 Infinity LC ultrahigh-performance liquid chromatography (UHPLC) (Agilent, CA, USA) HILIC column (Waters, MA, USA), and the samples were placed in an autosampler at 4 °C throughout the analysis. Continuous analysis of samples was performed in random order to avoid the effects of fluctuations in the instrument detection signal. An AB Triple TOF 6600 mass spectrometer (AB SCIEX, MA, USA) was used to collect the primary and secondary spectra of the samples.

### 5.9. Statistical Analyses

Data for blackberry are presented as the mean ± standard deviation (SD); data were obtained using the SPSS 24.0 statistical software program (SPSS 24.0, Chicago, IL, USA). One-way ANOVA was used to compare the mean values, and Duncan’s post hoc multiple comparisons were used to calculate the significance of differences in flavonoid, anthocyanin, and total phenolic contents and *RuFLS2* expression levels in different tissue parts (flowers, roots, stems, leaves, and fruiting branches) and fruits at different ripening periods (*p* < 0.05).

For the metabolome analysis of transgenic tobacco, unsupervised dimension reduction principal component analysis (PCA) was applied to all samples using the R package model (http://www.r-project.org/, accessed on 1 January 2022) to initially show the difference between groups of samples. Partial least squares discriminant analysis (PLS-DA) was applied to the control group using the R package ropls (http://www.r-project.org/, accessed on 1 January 2022) to better differentiate between the two groups by screening for variables related to the classification membership metabolomic signature [39]. Latent Structure Orthogonal Projection Discriminant Analysis (OPLS-DA) [40] was further used for cross-validation and permutation tests using the R package model (http://www.r-project.org/, accessed on 1 January 2022) in the control group. The validation of OPLS-DA models in metabolomics was performed with predictive power (Q^2^) values. Tests were carried out randomly 200 times to generate a distribution of R^2^’ values and Q^2^’ values.

## Figures and Tables

**Table 1:** Physicochemical properties of RuFLSs.

Name	Number of Amino Acids	Molecular Weight	Theoretical pI	Negatively Charged Residues (Asp + Glu)	Positively Charged Residues (Arg + Lys)	Instability Index (II)	Aliphatic Index	Hydropathicity
RuFLS1	339	39.03	5.72	42	32	36.45	81.36	−0.494
RuFLS2	331	37.30	5.44	44	33	34.70	88.91	−0.341

**Table 2:** Variation in flavonoid, phenol, and anthocyanin contents at different developmental stages in blackberry.

Fruit Color	Phenols (mg·g^−1^ FW)	Flavonoids (mg·g^−1^ FW)	Anthocyanins (mg·g^−1^ FW)
Green	40.68 ± 1.00a	11.50 ± 0.37a	0.02 ± 0.002d
Green–red	11.91 ± 1.05b	6.97 ± 0.11b	0.05 ± 0.01d
Red	3.28 ± 0.03c	1.81 ± 0.01c	0.39 ± 0.01c
Red–purple	2.35 ± 0.01c	1.80 ± 0.12c	0.93 ± 0.02b
Purple	3.10 ± 0.01c	1.95 ± 0.10c	2.60 ± 0.03a

Note: Different letters indicate significant differences between the different developmental stages of blackberry (*p* < 0.05).

**Table 3:** Main differentially expressed metabolites related to flavonoid biosynthesis in WT and RuFLS2-OX tobacco.

Pathway	Name	Fold Change
ko00941//Flavonoid biosynthesis	Phlorizin	−0.72
	Neohesperidin	−0.77
	Naringenin-7-*O*-glucoside	0.70
	(+)-catechin	−0.09
	Catechin gallate	−0.59
	Nobiletin	−0.48
ko00944//Flavone and flavonol biosynthesis	Apigenin 7-glucoside	0.61
	Kaempferol 3-*O*-rutinoside	0.58
	Luteolin 7-glucoside	−0.01
	Astragalin	2.74
	Quercitrin	0.46
	Quercetin 3-glucoside	−0.07
	Ligustroflavone	−0.60

## Data Availability

The data and materials supporting the conclusions of this study are included within this article.

## Supplementary Materials

The following supporting information can be downloaded at: https://www.mdpi.com/article/10.3390/ijms232214230/s1, Supplementary Table S1. Growth conditions for blackberry in a subtropical monsoon climatic zone; Supplementary Table S2. PCR primer information for the blackberry RuFLS2 gene and flavonoid biosynthesis pathway genes in WT and RuFLS2-overexpressing tobacco.
