# Supplementary material for: Overexpression of RuFLS2 Enhances Flavonol-Related Substance Contents and Gene Expression Levels
Source: Int J Mol Sci. 2022 Nov 17;23(22):14230. doi: 10.3390/ijms232214230 (PMC9699159; doi:10.3390/ijms232214230)
Supplement: Supplementary file 1 [file ijms-23-14230-s001.zip › ijms-2019947-supplementary.pdf]

Supplemental material

Supplementary Table S1. Growth conditions for blackberry in a subtropical monsoon climatic zone

Average temperature	Annual	15.6℃
	From June to August	27.1℃
Rainfall	Annual	1031.9 mm
	From June to August	435.6 mm
Major growth elements	Average sunshine	50%
	Soil pH	5.52
	Organic matter	18.67 g/kg
	Nitrogen	1.25 g/kg
	Phosphorus	4.83 mg/kg
	Potassium	94.21 mg/kg

Supplementary Table S2. PCR primer information for the blackberry *RuFLS2* gene and flavonoid biosynthesis pathway genes in WT and *RuFLS2*-overexpressing tobacco

Primer name	Primer sequence (5'-3')
RuFLS2-F	ATGGAGGTAGTGAGAGTTCAGGCG
RuFLS2-R	TTGTGGGATAGAGTTGAATTTGCG
RuFLS2-qRTF	AGCTGCCGGCCAAGTTCATC
RuFLS2-qRTR	GACCACGTCGTGTGGCTGAG
M13-F	TGTAAAACGACGGCCAGT
M13-R	CAGGAAACAGCTATGACC
Ru18S-F	ACGTCATCCTCCGGCAAAGC
Ru18S-R	ACGACGAAGCTCGCAAGTACAC
NtActin-F	CAAGGAAATCACCGCTTTGG
NtActin-R	AAGGGATGCGAGGATGGA
NtFLS-qF	AGGTGACACTATAGAATACACTTGGGCTTGGGTTAGAA
NtFLS-qR	GTACGACTCACTATAGGGAGAGGCCTTGGACTTCATTTG
NtF3H-qF	AGGTGACACTATAGAATATAAGGCTTCATCCATCCACC
NtF3H-qR	GTACGACTCACTATAGGGACAAAGTCATTTCCTCGCACA
